# Effects of bioflavonoid-containing mouth rinses on optical properties of tooth-coloured dental restorative materials

**DOI:** 10.1038/s41598-022-14254-2

**Published:** 2022-06-15

**Authors:** Tihana Divnic-Resnik, Jay Junyang Shen, Jim Vinh The Nguyen, Derek Weidi Lu, Vesna Miletic

**Affiliations:** The University of Sydney, Faculty of Medicine and Health, Sydney Dental School, 2 Chalmers Street, Surry Hills, 2010 Australia

**Keywords:** Biomaterials, Dental materials, Restorative dentistry

## Abstract

This study investigated differences in colour (ΔE_00_) and translucency parameter (ΔTP_00_) of nanofilled/microhybrid composites and a glass-ionomer cement following immersion in bioflavonoid (Citrox)- or chlorhexidine-based mouth rinses. Sixty disc-shaped specimens (N = 5/group) of Filtek Supreme (3M), Gradia Anterior (GC) and Fuji IX (GC) were exposed to Citrox/0.2%CHX (Perio+0.2, Curaprox), Citrox/0.09%CHX (Perio+0.09, Curaprox), 0.2%CHX (Savacol, Colgate-Palmolive) or distilled water by 2-min agitation daily for 28 days in an orbital shaker at 200 rpm at 37 °C. Colour recordings were performed using a clinical spectrophometer to obtain CIELab coordinates. General linear model, ANOVA, Tukey test (α = 0.05) and Pearson correlation test were used to analyse data. ΔE_00_ ranged between 0.33 (Gradia_Savacol_T28) and 6.35 (Fuji_Savacol_T28) (*p* < 0.001). ΔTP_00_ ranged between 0.36 (Fuji_ Perio+0.2) and 1.73 (Fuji_Savacol) (*p* < 0.05). Savacol resulted in higher ΔE_00_ of Filtek and Fuji and ΔTP_00_ of Filtek than Perio+0.09 and Perio+0.2 (*p* = 0.005). Perio+0.09 and Perio+0.2 resulted in higher ΔE_00_ at T7 than T28 (*p* < 0.05). There was no correlation between ΔTP_00_ and ΔE_00_ (r = 0.445, *p* = 0.147). Generally, Perio+0.2 and Perio+0.09 mouth rinses produced similar or lower ΔE_00_ and ΔTP_00_ than Savacol. GIC Fuji showed higher ΔE_00_ and similar or higher ΔTP_00_ than composites Filtek and Gradia. ΔE_00_ in all materials decreased in Perio+0.2 and Perio+0.09 over time.

## Introduction

Chlorhexidine (CHX) is considered the “gold standard” antiseptic for primary and secondary prevention of gingivitis and periodontitis as an adjunct to mechanical plaque control^1^. Commonly, it is used as antibacterial mouth rinse at various concentrations twice a day for up to four weeks. Although CHX exhibits broad antimicrobial spectrum and has outstanding substantivity, serious adverse effects of CHX have been increasingly recognised, such as the risk of type I and IV hypersensitivity^2^ and increased antimicrobial resistance^3,4^. Another major drawback of CHX is its ability to stain teeth^5,6^ and aesthetic restorative materials, namely composite resins^7^, zirconia and feldspathic ceramics^8^. Attempts with anti-discolouration systems have been made to reduce the staining potential of CHX, but with limited success^9^.

Alternative avenues have been explored and natural substances assessed for their potential to supplement or replace CHX in dental and medical products. Bioflavonoids are phenolic compounds with recognized diverse biological and health promoting effects such as anti-inflammatory, immunomodulatory, antioxidative and antimutagenic properties^10^. They also may increase vascular resistance and support wound healing^11^. Fruits, vegetables, nuts, seeds and spices are most common sources of bioflavonoids, with the largest amounts found in citrus fruits, blueberries, blackberries, onions, peppers, oregano and parsley^12^.

Citrox is a soluble formulation that contains a combination of nine bioflavonoids and organic acids, and possesses strong antimicrobial, anti-inflammatory and antioxidative properties^13,14^. Several Citrox formulations at various inhibitory concentrations have been tested for oral application, showing that Citrox BC30 inhibited growth of the majority of oral pathogens at concentrations between 1–2% (v/v)^15^.

To this end, reducing concentration of CHX and supplementing it with another potential antiseptic, may result in reduced side effects, whilst maintaining antimicrobial properties. Initial in vitro studies have shown enhanced antimicrobial effect of Citrox 1% in combination with various concentrations of CHX against common oral pathogens cultured planktonically and as cariogenic and perio-pathogenic biofilms^14,16^.

Tooth colour is known to have a strong impact on patient’s self-esteem and quality of life^17^. Another aspect of colour and appearance is the impact on longevity of dental restorations as surface staining is an important clinical criterion for their evaluation^18^. Following primary colour attributes, translucency is viewed as the most important optical property and one of fundamental factors affecting aesthetic appearance of dental restorations^19^. Optical properties may be affected by extrinsic and intrinsic differences in materials, such as surface roughness^20^ and sorption^21,22^.

Recently published visual thresholds define colour and translucency match/mismatch in dentistry^23^. CIEDE2000 visual thresholds for colour differences (ΔE_00_) are defined as excellent match (ΔE_00_ ≤ 0.8), acceptable match (0.8 < ΔE_00_ ≤ 1.8), mismatch type [a] (1.8 < ΔE_00_ ≤ 3.6), mismatch type [b] (3.6 < ΔE_00_ ≤ 5.4) and mismatch type [c] (ΔE_00_ > 5.4). Similarly, differences in translucency parameter (ΔTP_00_) are defined as excellent match (ΔTP_00_ ≤ 0.6), acceptable match (0.6 < ΔTP_00_ ≤ 2.6), mismatch type [a] (2.6 < ΔTP_00_ ≤ 5.2), mismatch type [b] (5.2 < ΔTP_00_ ≤ 7.8) and mismatch type [c] (ΔTP_00_ > 7.8)^23^.

Colour stability of microhybrid and nanohybrid/nanofilled composites has been tested in a variety of clinically relevant scenarios, including the effects of surface coating^24^, underlying dentine replacement material^25^, bleaching agents^26^, mouth rinses^27,28^, coloured food^29^ and beverages^30–32^. Similarly, glass ionomer cements (GICs) were tested for staining after exposure to coloured beverages^27^ but no data was found for mouth rinses. A recent meta-analysis has reported on staining potential of a number of commercially available mouthwashes on dental composites^33^. However, literature lacks data on the effect of CHX and, particularly, bioflavonoid complex (Citrox)-containing mouth rinses on optical properties (colour and translucency) of composites and GICs for direct restorations.

The aim of the study was to determine ΔE_00_ and ΔTP_00_ of a nanofilled and microhybrid composite and a reinforced GIC following immersion in bioflavonoid-containing (Citrox/CHX) mouth rinses or CHX control mouth rinse. The research hypotheses were that: (1) bioflavonoid-containing mouth rinses and CHX control had different effects on colour of evaluated restorative materials at several time intervals of 28-day exposure; (2) bioflavonoid-containing mouth rinses and CHX control had different effects on translucency of evaluated restorative materials after 28 days of exposure and 3) there is correlation between ΔE_00_ and ΔTP_00_ of the tested materials after immersion in mouth rinses.

## Results

Table 1 shows the summary of GLM analysis for factors “mouth rinse”, “material” and “time”. Both Perio+ mouth rinses resulted in lower ΔE_00_ than Savacol and water, with Perio+0.09 resulting in significantly lower ΔE_00_ than Perio+0.2 (*p* < 0.05). Savacol showed the greatest ΔE_00_ of the tested solutions (p < 0.05). The recorded ΔE_00_ for the tested materials were in the following order: Fuji > Filtek > Gradia (*p* < 0.05). There was no significant difference in discoloration after 7, 21 and 28 days (*p* > 0.05). Interaction of factors “mouth rinse”, “material” and “time” was significant (*p* < 0.05).

**Table 1:** Summary of GLM analysis of ΔE_00_ data for factors “mouth rinse”, “material” and “time”.

	N	Mean	Grouping
**Mouth rinse**			
Savacol	180	3.7	A
Water	180	2.4	B
Perio+0.2	180	1.7	C
Perio+0.09	180	1.3	D
**Material**			
Fuji	240	3.5	A
Filtek	240	2.2	B
Gradia	240	1.1	C
**Time**			
21 days	180	2.6	A
28 days	180	2.4	A
7 days	180	2.1	AB
14 days	180	2.0	B

Means that do not share a letter are significantly different. Grouping information using Tukey method and 95.0% confidence.

*N* number of observations, *mean* mean ΔE_00_ of each group within each factor.

Further analysis within factors is presented in Table 2. Comparing time intervals for each material-mouth rinse combination, significantly higher ΔE_00_ of Filtek and Gradia were recorded at T7 than T28 in all mouth rinses (*p* < 0.05) except for Filtek in Perio+0.2 (*p* = 0.221). Savacol resulted in significantly higher ΔE_00_ of Fuji at T28 than T7 (*p* = 0.008). Both Perio+ mouth rinses resulted in lower ΔE_00_ of Fuji at T28 than T7 but the difference was statistically significant for Perio+0.2 (*p* = 0.019). Water resulted in similar ΔE_00_ of Fuji (*p* = 0.269) and Gradia (*p* = 0.251) at T7 and T28 and lower ΔE_00_ of Filtek at T28 (*p* < 0.05).

**Table 2:** Mean (SD) values of colour difference (ΔE_00_) of the tested materials following exposure to mouth rinses at different time intervals.

Material	Mouth rinse	Time			
		T7	T14	T21	T28
Filtek	Perio+0.09	1.39 (0.07) A,c	0.54 (0.13) C,b	1.25 (0.27) A,c	0.73 (0.11) B,a,*,#
	Perio+0.2	2.26 (1.07) A,b	2.01 (1.26) A,a	1.84 (1.21) A,b	1.48 (0.83) A,b,*
	Savacol	4.19 (0.09) A,a	2.58 (0.22) C,a	4.07 (0.24) A,a	3.75 (0.12) B,c,*
	Water	2.45 (0.19) A,b	2.17 (0.15) B,a	2.31 (0.18) AB,b	1.96 (0.14) C,d,*
Gradia	Perio+0.09	0.93 (0.30) A,b	0.68 (0.17) B,c	0.53 (0.30) B,c	0.64 (0.19) B,a,#
	Perio+0.2	1.77 (0.25) A,a	1.04 (0.27) B,c	1.13 (0.36) B,b	1.04 (0.29) B,b,*
	Savacol	0.52 (0.15) A,c	0.60 (0.23) A,c	0.61 (0.15) A,c	0.33 (0.12) B,c,#
	Water	2.00 (0.19) A,a	1.97 (0.42) A,a	1.88 (0.22) A,a	1.80 (0.16) A,d,*
Fuji	Perio+0.09	1.43 (0.52) A,b	1.11 (0.41) A,c	1.69 (0.41) A,c	1.09 (0.40) A,c,*
	Perio+0.2	1.99 (1.56) A,ab	1.42 (0.61) A,c	1.84 (0.25) A,bc	1.51 (0.83) A,c,*
	Savacol	2.90 (0.46) A,a	4.70 (0.93) AB,a	6.11 (1.45) AB,a	6.35 (1.70) B,a, +
	Water	2.70 (2.10) A,ab	2.57 (1.30) A,b	3.64 (2.01) A,b	3.46 (1.81) A,b,*

Upper case letters—rows comparing times within each material-mouth rinse; Lower case letters—columns comparing mouth rinses within each material-time; Symbols—columns comparing materials at T28 within each mouth rinse.

Comparing mouth rinses at different material-time combinations, Savacol produced significantly higher ΔE_00_ of Filtek and Fuji than other mouth rinses (*p* < 0.05) for the same time interval, except Filtek at T14 and Fuji at T7 (*p* > 0.05). Conversely, ΔE_00_ of Gradia was significantly lower in Savacol compared to Perio+ mouth rinses at T7 and T28 (*p* < 0.05) and comparable to them at T14 and T21. Perio+0.09 resulted in significantly lower ΔE_00_ of Filtek and Gradia than Perio+0.2 for the same time interval (*p* < 0.05), except in Gradia at T14 which difference did not reach statistical significance. Perio+0.09 resulted in lower mean ΔE_00_ values in the Fuji group compared to Perio+0.2 but this difference did not reach statistical significance (*p* > 0.05). Water produced significantly higher ΔE_00_ of Gradia than Savacol and Perio+ mouth rinses at all times (*p* < 0.05) except Perio+0.2 at T7.

Comparing materials within each mouth rinse at T28, Savacol resulted in significant differences in ΔE_00_ in the following order: Fuji > Filtek > Gradia (*p* < 0.001). Perio+0.2 showed no differences in ΔE_00_ of the tested materials (*p* = 0.510). Perio+0.09 produced significantly higher ΔE_00_ of Fuji than Gradia (*p* = 0.049) with Filtek being in between the other two materials. No significant differences in ΔE_00_ of the tested materials were found in water, even with ln and sqrt transformations, despite Fuji showing considerably higher mean ΔE_00_ than Filtek and Gradia (*p* = 0.053).

**Figure 1:**
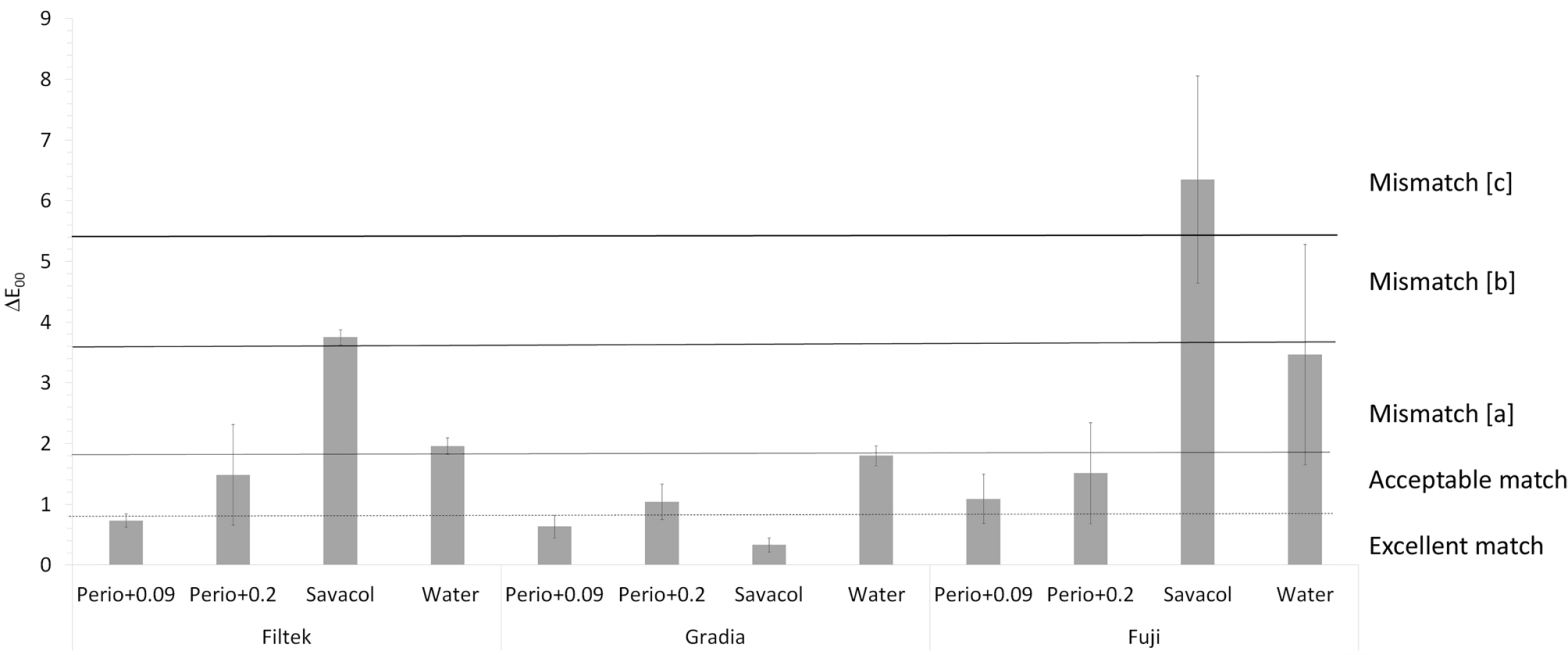
CIEDE2000 colour differences (ΔE_00_) for tested materials^23^. Excellent match (ΔE_00_ ≤ 0.8), acceptable match (0.8 < ΔE_00_ ≤ 1.8), mismatch type [a] (1.8 < ΔE_00_ ≤ 3.6), mismatch type [b] (3.6 < ΔE_00_ ≤ 5.4) and mismatch type [c] (ΔE_00_ > 5.4).

Figure 1 presents final ΔE_00_ data, corresponding to T0-T28, compared to CIEDE2000 visual thresholds. Perio+0.09 induced ΔE_00_ of Filtek and Gradia in the excellent match range and acceptable match in the Fuji group. Perio+0.2 resulted in ΔE_00_ of all materials that corresponded to acceptable match. Conversely, CHX-based control (Savacol) produced ΔE_00_ of Gradia in the excellent match range whilst ΔE_00_ of Filtek and Fuji corresponded to mismatch [b] and mismatch [c], respectively.

GLM analysis of ΔTP_00_ showed significant differences for factor “mouth rinse” (*p* = 0.006) but no differences for factor “material” (*p* = 0.060). However, interaction of the two factors was significant (*p* = 0.013).

Further analysis within factors is presented in Table 3. Comparing mouth rinses within each material, Savacol resulted in significantly higher ΔTP_00_ of Filtek than other mouth rinses (*p* = 0.005). There were no significant differences between mouth rinses in ΔTP_00_ of Gradia (*p* = 0.073) and Fuji (p = 0.086). Comparing materials within each mouth rinse, there were no significant differences in ΔTP_00_ among the tested materials after exposure to Savacol (*p* = 0.554) and water (*p* = 0.732). Fuji showed significantly higher ΔTP_00_ than other materials after exposure to Perio+0.09 (*p* = 0.006) whilst the same was true for Gradia after exposure to Perio+0.2 (*p* = 0.003).

**Table 3:** Mean (SD) values of translucency parameter differences (ΔTP_00_) after 28 days of exposure to mouth rinses.

Material	Mouth rinse	ΔTP_00_
Filtek	Perio+0.09	0.39 (0.45) B,a
	Perio+0.2	0.49 (0.44) B,b
	Savacol	1.39 (0.38) A,a
	Water	0.62 (0.33) B,a
Gradia	Perio+0.09	0.72 (0.22) A,a
	Perio+0.2	1.37 (0.43) A,a
	Savacol	1.17 (0.56) A,a
	Water	0.75 (0.42) A,a
Fuji IX	Perio+0.09	1.72 (0.75) A,b
	Perio+0.2	0.36 (0.11) A,b
	Savacol	1.73 (1.22) A,a
	Water	0.87 (0.68) A,a

Upper case letters—columns comparing mouth rinses within each material.

Lower case letters—columns comparing materials within each mouth rinse.

**Figure 2:**
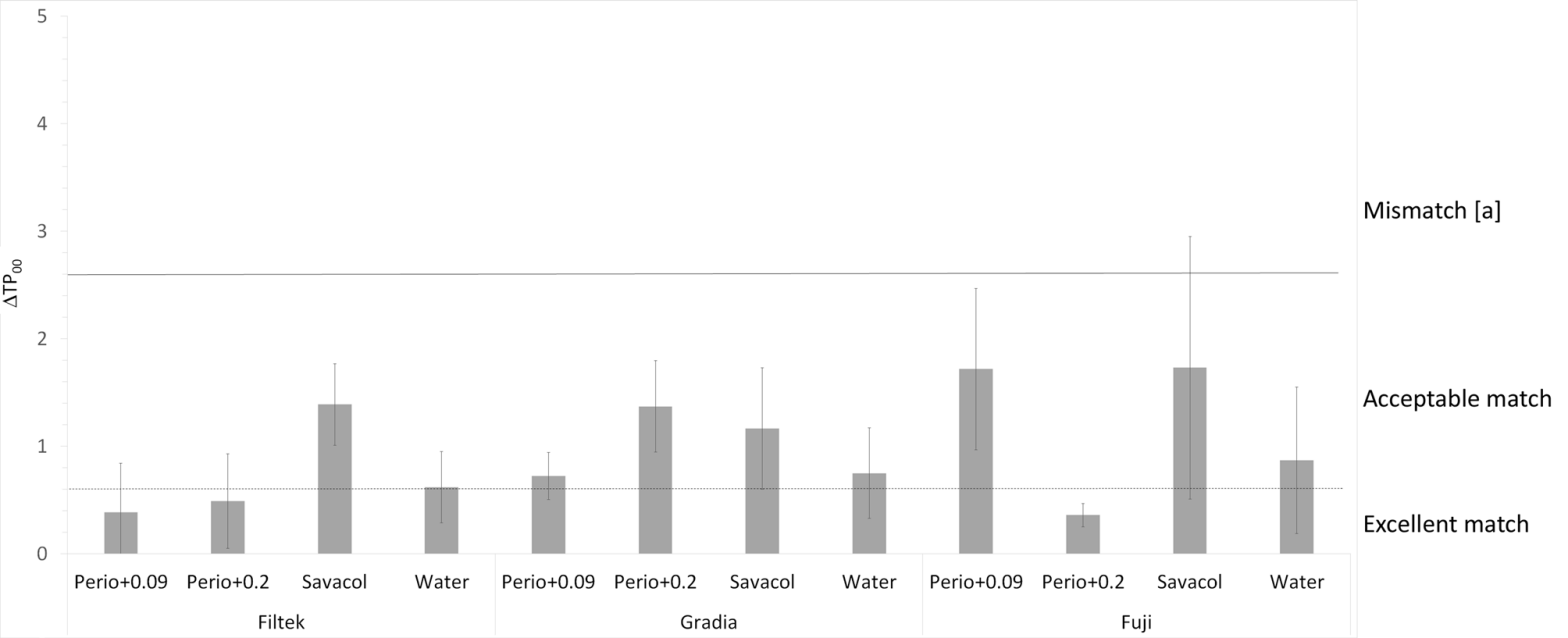
CIEDE2000 translucency parameter differences (ΔTP_00_) compared with literature data for visual thresholds for tooth-coloured restorative materials^26^. Excellent match (ΔTP_00_ ≤ 0.6), acceptable match (0.6 < ΔTP_00_ ≤ 2.6), mismatch type [a] (2.6 < ΔTP_00_ ≤ 5.2).

Figure 2 presents ΔTP_00_ data compared to CIEDE2000 visual thresholds. All groups showed ΔTP_00_ values that were excellent or acceptable match. CHX-based control (Savacol) produced acceptable match of ΔTP_00_ in all tested materials. Perio+0.2 resulted in excellent match ΔTP_00_ in Filtek and Fuji groups and acceptable match in Gradia. Perio+0.09 was associated with excellent match ΔTP_00_ in Filtek and acceptable match in Gradia and Fuji.

No correlation was found between ΔE_00_ and ΔTP_00_ after 28 days of exposure to mouth rinses (Pearson’s r = 0.445, *p* = 0.147).

## Discussion

Statistically significant colour differences were found among the tested materials following exposure to tested mouth rinses at different time intervals. Statistically significant differences in translucency among the materials were observed after 28 days of exposure. Therefore, both the first and second hypotheses were accepted. No significant correlation was found between ΔE_00_ and ΔTP_00_ of the tested materials after immersion in mouth rinses, hence the third hypothesis was rejected.

Compared to Savacol (control), Perio+0.09 and Perio+0.2 induced similar or lower ΔE_00_ in nanofilled composite Filtek Supreme and reinforced, conventional glass ionomer Fuji IX. In addition to bioflavonoids, citric and ascorbic acid, Perio+0.09 and Perio+0.2 contain polyvinylpyrrolidone/vinyl acetate (PVP-VA) copolymer, known for creating transparent films which adhere to various substrates, including glass and plastics. Lower staining capacity of Perio+0.09 and Perio+0.2 mouth rinses could be associated with PVP-VA copolymer protective film on material specimens as well as lower concentration of CHX (Perio+0.09). It was previously shown that surface protection of composite/glass ionomers with nanofilled coating reduces water sorption and internal staining^24^. PVP-VA copolymer in Citrox-containing mouth rinses could have a similar protective effect. It is unknown whether citrus bioflavonoids have an anti-staining effect on restorative materials.

The present findings showed initial ΔE_00_ at T7 to be higher than at T28 for Perio+0.09 and Perio+0.2 mouth rinses. Protective PVP-VA coating may also be the reason for a different staining pattern in composites (and glass ionomer), *i.e.* the lack of progressive increase in staining over time, which was previously reported in other staining scenarios^30,31^. It is generally known that staining of dental restorative materials has an extrinsic and intrinsic component, the former occurring due to pigment adsorption on material surface and the latter due to absorption of pigments and internal structural changes in the polymer. Increased development of the protective PVP-VA coating with subsequent specimen immersions in Perio+0.09 and Perio+0.2 may prevent further ingress of the staining medium. Another potential mechanism is the wash-out of loosely bound surface pigments during subsequent shaking cycles.

The ability of Savacol to limit or reduce staining over time was not as pronounced as Perio+0.09 and Perio+0.2 mouth rinses in Filtek and Gradia, and completely lacked in Fuji, which showed progressive increase in ΔE_00_ with the highest values detected at the final observation period. A similar effect of other CHX-based mouth rinses was previously reported for 7 and 14 days of exposure of microhybrid/nanofilled composites from the Filtek group^7^. Savacol contains glycerine, suggesting a limited film forming capacity than PVP-VA in Perio+0.09 and Perio+0.2. The idea that a surface coating may have a protective effect against staining is supported by a recent meta-analysis showing that the addition of an anti-discoloration system to CHX mouth rinses reduces tooth surface discoloration in non-brushing studies whilst the same effect was not observed in brushing studies^9^.

The effect of Savacol was strongly dependent on the material, Gradia showing substantial resistance to staining in Savacol than Perio+0.09 and Perio+0.2 mouth rinses. Gradia was generally less susceptible for staining in the tested mouth rinses than Filtek and Fuji. Higher staining of Filtek than Gradia in the present study could be due to greater pigment absorption of Filtek than Gradia. Greater volumetric changes were previously reported for Filtek than Gradia over 7 weeks of storage in water^34^ indicating greater susceptibility of Filtek for ingress of water and water-based media such as mouth rinses.

The present results showed higher staining for glass ionomer Fuji than composites Filtek and Gradia. This finding may be explained by generally greater sorption of glass ionomers compared to composites, as was previously reported^22^. Increased sorption leads to intrinsic staining due to pigment absorption and hydrolytical changes in the polyacid matrix. No GC coat was used in the present study to simulate conditions in which the material is exposed to the oral environment after the protective coating, initially placed to prevent water disbalance, is worn off. Another contributing factor to higher staining of glass ionomer Fuji compared to composites Filtek and Gradia is lower polishability of glass ionomers than composites. Glass ionomers tend to have higher surface roughness and more generalized surface irregularities than composites^20^. Glass ionomer matrix is based on loosely bound cation cross-linked polyacid molecules and is more likely to have larger filler particles dislodged during polishing compared to more efficient silane binding of resin matrix and filler particles in composites. Surface irregularities serve as pigment adsorption nuclei leading to increased surface staining.

Differences in ΔTP_00_ of the tested materials following exposure to mouth rinses were both material- and medium-dependent. ΔTP_00_ did not follow the same pattern as ΔE_00_ and no correlation between the two properties was established. TP is expressed as the relative amount of light passing through material and obtained by calculating colour difference of specimen against the white and black backgrounds^35^. Translucency is related to two aspects of light interaction with material (absorption and scattering) and factors such as surface topography, filler type and volume and refractive index mismatch between fillers and resin matrix have a significant effect^19^. Refractive index mismatch is known to change during polymerization as monomers convert to polymers but also after aging due to hydrolytical changes^16^. The present results indicate unpredictable effects of mouth rinses on ΔTP_00_ of restorative materials, that are both medium- and material-dependent.

Potential mechanisms of CHX staining of teeth have been suggested: (1) the Maillard reaction between sugars and proteins in the biofilm catalysed by CHX resulting in the formation of coloured pigments melanoidins; (2) Protein denaturation by CHX and the formation of organic yellow–brown ferric sulphides and (3) CHX and pigment interaction from coloured food and beverages^6,9^.

CHX staining mechanisms of composites and glass ionomers have not been elucidated. The same mechanisms previously suggested for tooth staining may be involved in material staining by CHX in the oral environment. However, in the present study, neither of the three mechanisms could be associated with material staining as no biofilm was used, there was no source of protein, and no colour food/beverages were used. Staining of restorative materials could also be related to the formation of pigments following chelation of CHX and inorganic elements in the materials. Namely, high staining of glass ionomer Fuji could be related to the interaction between CHX and Fuji’s iron oxides.

ΔE_00_ observed in the present study was interpreted based on literature data on CIEDE2000 visual thresholds^23^. Perio+0.09 resulted more frequently in ΔE_00_ of tested materials in the excellent match range than Perio+0.2, which resulted in acceptable match. Conversely, Savacol produced ΔE_00_ of Gradia in the excellent match range whilst ΔE_00_ of Filtek and Fuji corresponded to mismatch [b] and mismatch [c], respectively. A previous study on composites from the Filtek group ^7^ exposed to various CHX-based mouth rinses showed ΔE_00_ to be mostly an acceptable match, however the exposure of 14 days was shorter than in the present study. Even shorter exposure of ceramics to CHX-based mouth rinse in another study showed ΔE_00_ to be an excellent match^8^, likely due to material type and shorter exposure compared to the present study. ΔE_00_ of Gradia and Filtek in water were on the threshold between acceptable match and mismatch [a], respectively, and well in the mismatch [a] region in the Fuji group. The present results indicate that Perio+0.2 and Perio+0.09 induce minor to moderate changes in colour of restorative materials that are below the perceptibility threshold or within the acceptability limit. There is a dose-dependent relationship between the CHX concentration in these mouth rinses, the higher CHX concentration the greater ΔE_00_ of restorative materials.

Similarly, ΔTP_00_ observed in the present study were interpreted based on literature data on CIEDE2000 visual thresholds^23^. All groups showed ΔTP_00_ values that were excellent or acceptable match. The present results indicate that exposing restorative materials to mouth rinses, in general, results in minor changes in translucency that are below the perceptibility threshold or within the acceptability limit.

Based on the present results, it is concluded that:

- The effects of mouth rinses on ΔE_00_ were medium-, material- and time-dependent. Perio+0.09 and Perio+0.2 produced lower ΔE_00_ of Filtek Supreme and Fuji IX than Savacol. Savacol induced the lowest ΔE_00_ of Gradia among the tested media.
- Glass ionomer Fuji IX showed greaterΔE_00_ than BisGMA-based nanofilled composite Filtek Supreme and UDMA-based microhybrid composite Gradia Anterior following exposure to mouth rinses.
- Perio+0.09 and Perio+0.2 showed similar or lower ΔE_00_ from 7 to 28 days of exposure.
- The effects of mouth rinses on ΔTP_00_ were medium- and material-dependent. Perio+0.09 and Perio+0.2 produced lower ΔTP_00_ of Filtek than Savacol whilst no differences were observed between mouth rinses in relation to ΔTP_00_ of Gradia and Fuji. Fuji IX showed significantly higher ΔTP_00_ than other materials after exposure to Perio+0.09 whilst the same was true for Gradia after exposure to Perio+0.2.
- No correlation was found between ΔTP_00_ and ΔE_00_ after 28 days of exposure to mouth rinses.

## Methods

### Specimen preparation

Sixty disc-shaped specimens (N = 5/group) were fabricated using the aesthetic dental restorative materials listed in Table 4. All materials were shade A2.

**Table 4:** Details of tested materials and mouth rinses.

Material	Composition	Manufacturer
Filtek Supreme XTE (Filtek)	Silane treated ceramic, silica and zirconia, BisGMA, BisEMA-6, UDMA, PEGDMA, TEGDMA, Phenyl bis*(2,4,6-trimethyalbenzoyl)-phosphine oxide	3M, St. Paul, MN
Gradia Direct Anterior (Gradia)	UDMA, 2,2-dimethyl-1,3-propanediyl bismethacrylate, Propylidynetrimethyl trimethacrylate, 2,2'-dimethyl-2,2'-azodipropiononitrile, Butylated hydroxytoluene, Bornane-2,3-dione, 6-tert-butyl-2,4-xylenol, 2-(2H-benzotriazol-2-yl)-p-cresol, 3-trimethoxysilylpropyl methacrylate, 3-methylbutyl 4-(dimethylamino)benzoate, Glass oxide, SiO_2_	GC Corp, Tokyo, Japan
Fuji IX Extra GP (Fuji)	Polyacrylic acid, Tartaric acid, Water, Glass oxide, Mixture of iron oxides	
**Mouth rinse**		
PerioPlus^+^ Forte (Perio+0.2)	0.2% Chlorhexidine-digluconate, Citrox/P complex, xylitol, polylysine, polysorbate 20, aroma, phenoxyethanol, PVP/VA copolymer, sucralose, cetylpiridinium chloride, citric acid, citrus aurantium amara fruit extract, glycerine, sodium hydrochloride, sodium chloride, water Alcohol free	Curaprox, Kriens, Switzerland
PerioPlus^+^ Regenerate (Perio+0.09)	0.09% Chlorhexidine-digluconate, Citrox/P complex, hyaluronic acid, propylene glycol, sodium gluconate, xylitol, PEG-40 hydrogenated castor oil, polysorbate 20, sodium hyaluronate, aroma, cyclodextrin, sucralose, cetylpyridinium chloride, disodium phosphate, sodium phosphate, citric acid, citrus aurantium amara fruit extract, glycerine, polylysine, sodium chloride, water Alcohol-free	
Savacol (Savacol)	0.2% (2 mg/ml) Chlorhexidine, glycerine, sorbitol, water, Alcohol-free	Colgate-Palmolive, New York, NY

*BisGMA* bisphenol A diglycidyl ether dimethacrylate, *BisEMA-6* bisphenol A polyethylene glycol diether dimethacrylate, *UDMA* diurethane dimethacrylate, *PEGDMA* polyethylene glycol dimethacrylate, *TEGDMA* triethylene glycol dimethacrylate, *PVP-PA* polyvinylpyrrolidone/vinyl acetate.

Composite resin and GIC specimens were prepared by filling standardized polyvinylsiloxane moulds (Affinis heavy body 6520), 8 mm in diameter and 2 mm thick, held on a glass slide. Composite specimens were covered with a transparent polyethylene terephthalate strip (Mylar, Henry Schein, Melville, NY, USA), pressed with another glass slide to extrude excess materials and light-cured through the strip with a LED light-curing unit (Elipar™ DeepCure-S, 3M, St. Paul, MN), operating at an intensity of 1400–1500 mW/cm^2^ and wavelength of 430–480 nm. GIC specimens were pressed with a glass slide to extrude excess material and allowed to set for 6 min. Each disc-shaped specimen was wet-polished using resin finishers and polishers (Dentsply Sirona Enhance Finishing and Polishing System Kit) for 30 s. Prior to staring the experiment, specimens were immersed in distilled water and incubated at 37 °C for 24 h in a closed container to simulate the oral cavity.

Subsequently, specimens of each aesthetic material were randomly allocated to one of four groups according to staining solutions. Mouth rinses containing Citrox/0.2%CHX and Citrox/0.09% CHX were test solutions (Perio+0.09 and Perio+0.2, respectively), mouth rinse with 0.2% CHX with no additives (Savacol) was used as a positive control and distilled water as a negative control (Table 4).

Prior to the experiment, pH values of each of the solutions were measured using a pH meter *(PHM 83 AUTOCAL pH METER).* Average pH of PerioPlus mouth rinses was 7.22, Savacol 8.24 and distilled water 7.28, respectively.

Containers with specimens immersed in 50 ml of their respective solutions were placed on an orbital shaker (*Infors AG CH-4103 Bottmingen*) at 200 rpm at 37 °C and agitated for two minutes to mimic the effect of one-minute rinsing two times per day, according to manufacturer’s recommendations. Staining solutions were renewed daily. Following each cycle of rinsing procedure, specimens were incubated in distilled water at 37 °C for the rest of the day and overnight. The rinsing cycle was repeated daily for four weeks to simulate the use of mouth rinses as commonly prescribed during periodontal treatment.

### Colour measurements

CIELab colour coordinates were recorded to determine colour and translucency differences before and after specimen exposure to solutions. Following initial immersion in distilled water, baseline colour values (L*, a*, b*) were measured against a white and black background using a calibrated clinical spectrophotometer *(VITA EasyShade V, Zahnfabrik, Bad Säckingen, Germany)*. Colour measurements against the white background were recorded at baseline and after each full cycle corresponding of 7, 14, 21 and 28 days of clinical exposure (T7, T14, T21 and T28, respectively). The black background measurements were recorded at baseline and after 28 days (T28).

Colour differences (*ΔE*_*00*_*)* were calculated using CIEDE2000 formula^36^:

$$ \Delta E_{00} = \left[ {\left( {\frac{{\Delta L^{\prime } }}{{K_{L } S_{L} }}} \right)^{2} + \left( {\frac{{\Delta C^{\prime } }}{{K_{C } S_{C} }}} \right)^{2} + \left( {\frac{{\Delta H^{\prime } }}{{K_{H } S_{H} }}} \right)^{2} + R_{T} \left( {\frac{{\Delta C^{\prime } }}{{K_{C} S_{C} }}} \right)\left( {\frac{{\Delta H^{\prime } }}{{K_{H} S_{H} }}} \right)} \right]^{\frac{1}{2}} $$

Translucency values (TP_00_) were calculated using the following formula^35^:

$$ TP_{00} = \left[ {\left( {\frac{{L_{B}^{\prime } - L_{W}^{\prime } }}{{K_{L} S_{L} }}} \right)^{2 } + \left( {\frac{{C_{B}^{\prime } - C_{W}^{\prime } }}{{K_{C} S_{C} }}} \right)^{2 } + \left( {\frac{{H_{B}^{\prime } - H_{W}^{\prime } }}{{K_{H} S_{H} }}} \right)^{2 } + RT \left( {\frac{{C_{B}^{\prime } - C_{W}^{\prime } }}{{K_{C} S_{C} }}} \right)^{ } \left( {\frac{{H_{B}^{\prime } - H_{W}^{\prime } }}{{K_{H} S_{H} }}} \right)} \right]^{1/2} $$

where *∆L′, ∆C′* and *∆H′* are metric differences computed on the basis of the uniform colour space used in CIEDE2000 and *L’, C’ and H’* denote lightness, chroma and hue, respectively, against white (*_W_) and black (*_B_) backgrounds. SL, SC, SH adjust the total colour difference for variation in the location of the colour difference sample over the B and W backgrounds in L′, a′, b′ coordinates. The empirical terms *K*_*L*_*S*_*L*_*, K*_*C*_*S*_*C*_ and *K*_*H*_*S*_*H*_ are used for correcting (weighting) the metric differences to the CIEDE2000 differences for each coordinate. Parametric factors *K*_*L*_*, K*_*C*_ and *K*_*H*_ were set at 1. RT accounts for the interaction between chroma and hue differences in the blue region.

ΔE_00_ was determined for each measurement interval (T0-T7, T0-T14, T0-T21 and T0-T28). ΔTP_00_ was calculated as the difference between TP_00_ values measured initially (T0) and after final immersion period (T28).

### Statistical analysis

Data were statistically analysed in the software package Minitab 16 (Minitab Inc., State College, PA). Data were first checked for normality and equal variances as preconditions for parametric testing. If necessary, the appropriate data transformation was performed, *e.g.* log or sqrt to achieve normality and/or equal variances. Data were tested using general linear model (GLM) for factors “material”, “mouth rinse” and “time” with included factor interaction. In case of significant factor interaction, further analysis of variance (ANOVA) were performed within each factor. Tukey's post-hoc test was used for intergroup comparison. Pearson correlation was used to test the relationship between ΔE_00_ and ΔTP_00._ The level of significance was set at 0.05.

## Data Availability

All data are available from the corresponding author upon request.
